# 

**Published:** 1847-04

**Authors:** 


### EDITORIAL DEPARTMENT.

*National Medical Convention.*—The meeting of the National Convention, appointed to take place at Philadelphia, on the first Wednesday of May, 1847, is now near at hand. From our Exchange Journals, and other sources, we learn that delegates have been chosen from Medical Societies and Colleges very generally throughout the country. The prospect is, that most, if not all the States of the Union will be represented. Large delegations will doubtless attend from States contiguous, or connected by easy facilities of travelling, and if we may judge from various manifestations of interest in different and remotely separated quarters, as well as by the names of those who are known to have been elected delegates, the approaching Convention, in numbers, and in character, will be without a parallel not only in the past annals of medicine in this country, but in those of the next half century to come. The unexpectedly full attendance at the convention of 1846, the enthusiasm and harmony which pervaded that assemblage, the prudence and judgment which characterised its proceedings, removed, for the most part, an apathy and distrust, not to say suspicion, with which, by some, the movement was at first regarded. The profession seems now sufficiently united upon the practicability of the convention. That it will take place, and in its composition be all that could be desired, or at least, expected, is quite certain ; but it remains to be determined whether it will accomplish results commensurate with the elaborateness of the undertaking, and the sanguine expectations of those who have manifested most interest in its success. As the period is so near when this question will be practically settled, it would hardly be worth our while to enter upon a full discussion of it at this time. We are free to say, however, that we shall not only be disappointed, but greatly mistaken in our anticipations, if the approaching convention does not prove to be fraught with important influences upon the condition of medical science and the medical profession of this country. Our expectations from it, however, do not relate entirely to any action which may be had on all the particular subjects which will come before it, nor upon any immediate changes which it may endeavor to effect. We would take a broader view of its salutary operation than this. That it may do much which will prove immediately and palpably useful is quite probable, but the movement appears to us more especially important as a starting point for united zeal and organized effort among not a sectional portion, but, collectively, of the Medical Profession of our whole country. We should have but little hopes from the convention if we did not extend our ken beyond its formal proceedings ; but we look upon it as the beginning of a new order of things, in which our profession is to assume the guardianship of its character and interests, and is hereafter to legislate and act for itself. Although in many respects this meeting will be peculiarly interesting, and will probably be the occasion of a much larger assemblage than may again occur in the present generation, it will by no means be the conclusion of the matter. In some form or other the profession will continue to act, through its representatives, to perfect and perpetuate the advantages of a satisfactory organization. It is in this light we believe this convention promises to become an epoch which will never be lost sight of in the medical history of our country.

There is another aspect in which, as it seems to us, for the sake of justice to the subject, and to the profession of this country, the present movement should be regarded. It is not merely a scheme of a few individuals, devised to bring about a new state of feeling with respect to the objects which it proposes ; but it is the legitimate result of a prevailing sentiment, which has been for years developing and diffusing itself. We have certainly no disposition to depreciate the services of those who have originated the undertaking, and labored to promote it. They deserve thanks for their timely efforts ; but it is to be considered that the medical mind was, to a great extent, already prepared for united deliberations, and concerted action. The invitation emanating from our State Society, therefore, was warmly responded to by the profession in all the States. There were some exceptional instances of caviling, but the manner in which the project was generally received, showed that it harmonised with a pre-existing, widely prevalent sentiment. Had it not been so, such are the difficulties peculiarly operative in the way of assembling a large body of medical practitioners, that the plan must inevitably have fallen through ; and it is upon the same sentiment which secured the favorable reception of the project in the first instance, that chief reliance is still to be placed for the attainment of the beneficial ends of organized effort. The present wide spread interest, then, in medical reform, as it is called, is by no means a newly developed spirit. It is an abiding interest, which has long been felt, but which now, in consequence of its increased energy, and the force of various circumstances, finds louder utterance, and seeks to eventuate in action. The expression *Medical Reform* is calculated to convey an erroneous idea.—We are not prepared to say that there exists no necessity for reform, but we do not, on the other hand, think that the condition of the medical profession is so depressed, or degraded, as the constant application of the term to the present movement would seem to denote. The impression that Medicine has been retrograding as regards its position with the public, and the intrinsic character belonging to the body of its practitioners, we cannot but deem erroneous. So far from this, if we review the history of medical education for the last few years, and institute comparisons in the average attainment of the Medical Fraternity, we shall perceive constant advancement, in a ratio corresponding, in some measure at least, with the progressive improvement of medical science ; nor, shall we discover, that rampant empiricism, and the truculence of fanatical opposition, have made any considerable inroads in the confidence of community. The desire for union and co-operation for the promotion of scientific and professional objects, is, in our view, one of the encouraging and animating signs of the times : it is an indication of progress, not the evidence of a crisis in which some desperate measures are requisite in order to retrieve our waning fortunes.

By the tenor of the foregoing remarks our readers will perceive, that the first, and most important of the benefits which we anticipate from the Convention, is the institution of a permanent *National Medical Association* by the profession of the United States, "for the protection of their interests, for the maintenance of their honor and respectability, for the advancement of their knowledge, and the extension of their usefulness."* Although this is but one of the objects which will come before the Convention, and although the others are by no means of inconsiderable importance, and will doubtless not be overlooked, yet, in our opinion, the profession would have no cause of complaint if this alone were attained. A National Association satisfactorily organised, and commending itself to the favor of the Profession, will exert an incalculable influence in the promotion of medical science, and the elevation of the medical profession, by affording a focus for the concentration of the talent and industry which already exist in our ranks, by supplying those incentives to exertion derived from opportunities for honorable distinction, by facilitating division of labor, by providing means and appliances for scientific investigations which individual enterprise is not adequate to procure, and by developing in various modes enthusiastic zeal, and diligence of application in the cultivation of the several branches of Medical knowledge. This association possessing, as it ought to do, competent powers for the regulation of all matters pertaining to Medical education, ethics and polity, will be able to accomplish by gradual improvements, what could never be done by sudden and violent alterations. It will be the means of forming a national medical character, by which the profession of this country will be known and respected by our brethren and co-laborers in the universal republic of Science. We could easily amplify upon the numerous sources of benefit to which we have alluded, but we trust that an opportunity will soon be offered to resume the subject in announcing the creation of such an institution as the resolution of the Convention of 1846 contemplated. The committee appointed to report a plan of organization, will be prepared to

* The terse language of the first resolution adopted by the former Convention.

present to the Convention of 1847, matured results of long and earnest deliberation, which, we doubt not, will be acceptable both to the wisdom of the Convention, and to the profession at large.

The other topics committed by the last convention to committees to be reported upon at the coming convention, relate, for the most part, to the standard of requirements, medical and preliminary, for the insignia of doctorship, and to the formation of an uniform code of medical ethics. We need not comment on the interest and importance which appertain to these topics. That the convention will recommend improvements tending to raise the qualifications of medical graduates is altogether probable. This the predominant sentiment of the profession expects and demands. Upon what particular dispositions will be made of the different questions involved in this object, we will not indulge any speculations at this time.—We will only express the hope, that the convention will act here, as in all other matters, with prudent moderation, and in a spirit of conciliation and harmony; avoiding ultra or extreme measures which must necessarily be impracticable, and endeavoring to reconcile by judicious compromises discordant views and interests, should they be found to exist.

*Arrangements for the third volume of the Buffalo Medical Journal.*—With the next No. of this Journal closes the second year of its existence. Permanent arrangements have been made for its continuance. The publishing house of JEWETT, THOMAS & Co., have become joint proprietors of the work, and the editorial department remains as heretofore. We have to request that hereafter business communications, remittances, &c., shall be directed exclusively to the Publishers; letters relating to the editorial department, books, &c., may continue to be addressed to the editor.

It has been determined to reduce the price of the Journal for the coming year. It will be furnished for $2 50, in advance, and, to encourage subscriptions, five copies will be sent to respective addresses on payment of $10. As the amount of single subscriptions cannot very conveniently be enclosed in letters, advantage may be taken of the facilities afforded by the Post Office department, by sending a draft from the post master of the place where the subscriber resides. The course directed to be pursued in this mode of remittance is contained in regulations which we will cause to be published on the cover, for the accomodation of those who desire to subscribe; but if it be preferred to send it by letter, each subscriber may procure an associate, and send $5 00!

We believe we may say, that considering the size of the work, and its typographical execution, it will be the cheapest medical periodical published in the United States.

Owing to unavoidable circumstances, which, we are assured, will not hereafter occur, our monthly issues for the present year have not been so punctual always as we could have wished, and the no. for March in its mechanical execution, was inferior to the previous numbers. We feel, however, too deeply indebted to the liberality of our Publishers to complain, as they have printed the work much below the usual rates in consideration of the difficulties necessarily incident to the commencement of such an enterprise. Hereafter our patrons may expect our arrival within the first ten days of each month, and the present number may be taken as a specimen of the style which the work will preserve.

They who are indebted for the present volume of the Journal, are earnestly requested to forward the amount without delay.

EDITOR.

